# Impact of adenomyosis on perinatal outcomes: a large cohort study (JSOG database)

**DOI:** 10.1186/s12884-023-05895-w

**Published:** 2023-08-11

**Authors:** Hiroaki Komatsu, Fuminori Taniguchi, Tasuku Harada

**Affiliations:** 1 Department of Obstetrics and Gynecology, Tottori University School of Medicine, 36-1 Nishicho, Tottori prefecture, Tottori, 683-8504 Japan; 2 Tottori University, Tottori, Japan

**Keywords:** Placental abruption, Adenomyosis, Fertility, Fetal growth restriction, Japan, JSOG database, Miscarriage, Placenta accreta, Pregnancy, Uterine rupture

## Abstract

**Background:**

A previous study investigated the effect of adenomyosis on perinatal outcomes. Some studies have reported varying effect of adenomyosis on pregnancy outcomes in some patients and dependence on the degree and subtype of uterine lesions. To elucidate the impact of adenomyosis on perinatal outcomes.

**Methods:**

This large-scale cohort study used the perinatal registry database of the Japan Society of Obstetrics and Gynecology. A dataset of 203,745 mothers who gave birth between January 2020 and December 2020 in Japan was included in the study. The participants were divided into two groups based on the presence or absence of adenomyosis. Information regarding the use of fertility treatment, delivery, obstetric complications, maternal treatments, infant, fetal appendages, obstetric history, underlying diseases, infectious diseases, use of drugs, and maternal and infant death were compared between the groups.

**Results:**

In total, 1,204 participants had a history of adenomyosis and 151,105 did not. The adenomyosis group had higher rates of uterine rupture (0.2% vs. 0.01%, *P* = 0.02) and placenta accreta (2.0% vs. 0.5%, *P* < 0.001) than the non-adenomyosis group. A history of adenomyosis (odds ratio: 2.26; 95% confidence interval: 1.43–3.27; *P* < 0.001), uterine rupture (odds ratio: 3.45; 95% confidence interval: 0.89–19.65; *P* = 0.02), placental abruption (odds ratio: 2.11; 95% confidence interval: 1.27–3.31; *P* < 0.01), and fetal growth restriction (odds ratio: 2.66; 95% confidence interval: 2.00–3.48; *P* < 0.01) were independent risk factors for placenta accreta.

**Conclusion:**

Adenomyosis in pregnancies is associated with an increased risk of placenta accreta, uterine rupture, placental abruption, and fetal growth restriction.

**Trial registration:**

Institutional Review Board of Tottori University Hospital (IRB no. 21A244).

## Background

Adenomyosis is defined as the presence of endometrial glands and stroma within the myometrial layer. The true prevalence of adenomyosis is unknown, with overall reported rates of 1–70%, but it is thought to be 20%, specifically in women of reproductive age [1]. Previously, adenomyosis was considered to be associated with multiparity, not infertility. Since non-surgical diagnosis using ultrasound images and magnetic resonance imaging became possible, an association between adenomyosis and fertility or miscarriage was suggested [2].

During pregnancy, as it becomes difficult to evaluate the entire myometrial layer in the second trimester, the presence or absence of adenomyosis should be assessed before conception or in early pregnancy. A previous study investigated the effect of adenomyosis on perinatal outcomes [2]. Some studies have reported varying effect of adenomyosis on pregnancy outcomes in some patients and dependence on the degree and subtype of uterine lesions. The impact of endometriosis or uterine adenomyosis on perinatal outcomes was previously reported, using data from a large cohort of the Japan Environment and Children’s Study [3, 4]. The study concluded that adenomyosis increased the risk of placental abruption and fetal growth restriction (FGR), but the number of cases in the study, approximately 300, was not very large. No study has examined the effect of adenomyosis using large-scale data of > 1,000 individuals [5].

Here, we conducted a large-scale cohort study using the perinatal registry database of the Japan Society of Obstetrics and Gynecology (JSOG), aiming to elucidate the impact of adenomyosis on perinatal outcomes in another population.

## Methods

The perinatal registry database is a project managed by the JSOG that includes data from 408 facilities that provide perinatal care in Japan (107 university hospitals, 29 National Organization hospitals, 34 Red Cross hospitals, and 238 other facilities). The registry collects delivery data annually, and 95 of 110 general perinatal centers (86.3%) and 207 of 298 regional perinatal centers (69.5%) in Japan are included in the database. Patients who give birth after 22 weeks of gestation are enrolled in the database.

Data from 203,745 to 840,832 mothers (24.2%) who gave birth in Japan from January 2020 to December 2020 were included in this study. The participants were grouped into adenomyosis and non-adenomyosis groups based on their history of adenomyosis. Participant characteristics (age, pregnancy and delivery histories, medical history, and body mass index), fertility treatments, delivery data (delivery method, inclusion of hysterectomy, induction/labor acceleration, instrumental procedures, heart rate, and non-reassuring fetal status), obstetric complications, maternal treatments, infant data (gestational age, sex, height, weight, Apgar scores, and malformation), fetal appendage data, obstetric history, underlying diseases, infectious diseases, drugs used, maternal death data, and infant death data were compared between the groups.

This study was approved by the Institutional Review Board of Tottori University Hospital (IRB no. 21A244) and JSOG IRB committee (IRB no. 2021-17). All patients provided written informed consent following the institutional guidelines. All methods were carried out in accordance with relevant guidelines and regulations.

### Statistical analysis

Continuous data are presented as mean and standard deviation. Categorical data are presented as number and frequency. The Mann-Whitney U-test and chi-squared or Fisher’s exact test were used to compare the variables. A multivariate analysis using a logistic regression analysis was performed. Statistical significance was set at *P* < 0.05. All statistical analyses were performed using GraphPad Prism 8.3 software (GraphPad Software, Inc., La Jolla, CA, USA).

## Results

Of the 203,745 pregnancies in the database, there were 1,653 adenomyosis cases and 202,092 non-adenomyosis cases. Excluding cases with a history of cesarean section and twin pregnancies, this study examined 1,204 adenomyosis cases (adenomyosis group) and 151,105 non-adenomyosis cases (non-adenomyosis group).

Information on patient background is provided in Table 1.

**Table 1:** Patient characteristics

Variable		Adenomyosis (n = 1,204)	Non-adenomyosis (n = 151,105)	*P-*value
Age (years)		35	32	0.02
Number of pregnancies	P0	878 (72.9%)	86,455 (57.2%)	< 0.01
	P1 or more	326 (27.1%)	64,651 (42.8%)	
ART		349 (20.6%)	14,581 (9.6%)	< 0.01
Type of delivery	Spontaneous	588 (48.8%)	102,749 (67.9%)	< 0.01
	Instrumental	148 (12.3%)	14,069 (9.3%)	
	Selective Cesarean section	216 (18.0%)	21,708 (14.3%)	
	Emergency Cesarean section	252 (20.9%)	12,552 (8.3%)	
Gestational age at delivery (weeks)	22–36	193 (16.0%)	16,045 (10.6%)	< 0.01
	37–41	1,007 (83.6%)	134,683 (89.1%)	
	42+	3 (0.2%)	284 (0.2%)	
	Unknown	1 (0.08%)	91 (0.06%)	
Maternal morbidities	PROM	67 (5.6%)	9,031(6.3%)	< 0.01
	Threatened premature labor	233 (19.3%)	19,017 (12.5%)	< 0.01
	Uterine rupture	2 (0.2%)	27 (0.01%)	0.02
	Placental abruption	19 (1.6%)	1,449 (0.9%)	0.02
	HDP	121 (10.0%)	9,884 (6.5%)	< 0.01
Infant morbidity	FGR	64 (5.3%)	5,739 (3.8%)	< 0.01
Infant mortality		1 (0.1%)	11 (0.007%)	NS

Abbreviations: ART, assisted reproductive technology; PROM, premature rupture of membranes; HDP, Hypertensive disorders in pregnancy; FGR, fetal growth restriction; NS, not significant

The adenomyosis group was significantly older and had larger proportions of primiparas and pregnancies resulting from assisted reproductive technology (ART) than the non-adenomyosis group (adenomyosis vs. non-adenomyosis; 20.6% vs. 9.6%, *P* < 0.01). Further, the adenomyosis group had higher rates of cesarean Sect. (20.9% vs. 8.3%, *P* < 0.01) and premature deliveries (10.6% vs. 9.6%, *P* < 0.01) than the non-adenomyosis group. Additionally, the adenomyosis group had higher rates of premature delivery (19.3% vs. 12.5%, *P* < 0.01), uterine rupture (0.2% vs. 0.01%, *P* = 0.02), placental abruption (1.6% vs. 0.9%, *P* = 0.02), preeclampsia (10.0% vs. 6.5%, *P* < 0.01), and intrauterine growth restriction (5.3% vs. 3.8%, *P* < 0.01) than the non-adenomyosis group.

We then examined placental malposition. The adenomyosis group had significantly higher rates of placental malposition (total placenta previa, edge placenta previa, partial placenta previa, and low placenta) and placenta accreta (2.0% vs. 0.5%, *P* < 0.01) than the non-adenomyosis group (Table 2).

**Table 2:** Frequencies of abnormal placenta

		Adenomyosis (n = 1,204)	Non-adenomyosis (n = 151,105)	P-value
Abnormal placenta	Total placenta previa	37 (3.7%)	888 (0.5%)	< 0.01
	Edge placenta previa	16 (0.9%)	884 (0.5%)	< 0.01
	Partialplacenta previa	11 (1.3%)	298 (0.1%)	< 0.01
	Low placenta	31 (2.5%)	1,509 (0.9%)	< 0.01
	Placenta accreta	25 (2.0%)	822 (0.5%)	< 0.01

**Fig. 1:**
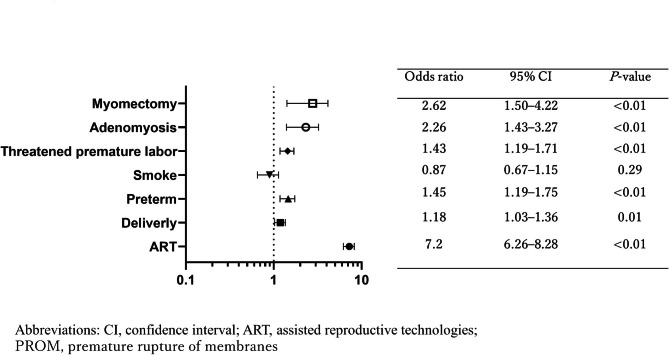
Risk factors for placenta accreta. A multivariate analysis identified ART pregnancies (odds ratio (OR): 7.20; 95% confidence interval (CI): 6.26–8.28; P < 0.001), complication of adenomyosis (OR: 2.26; 95% CI: 1.43–3.27; P = 0.001), and history of uterine myomectomy (OR: 2.62; 95% CI: 1.5–4.22; P < 0.01) as independent risk factors for placenta accreta

**Fig. 2:**
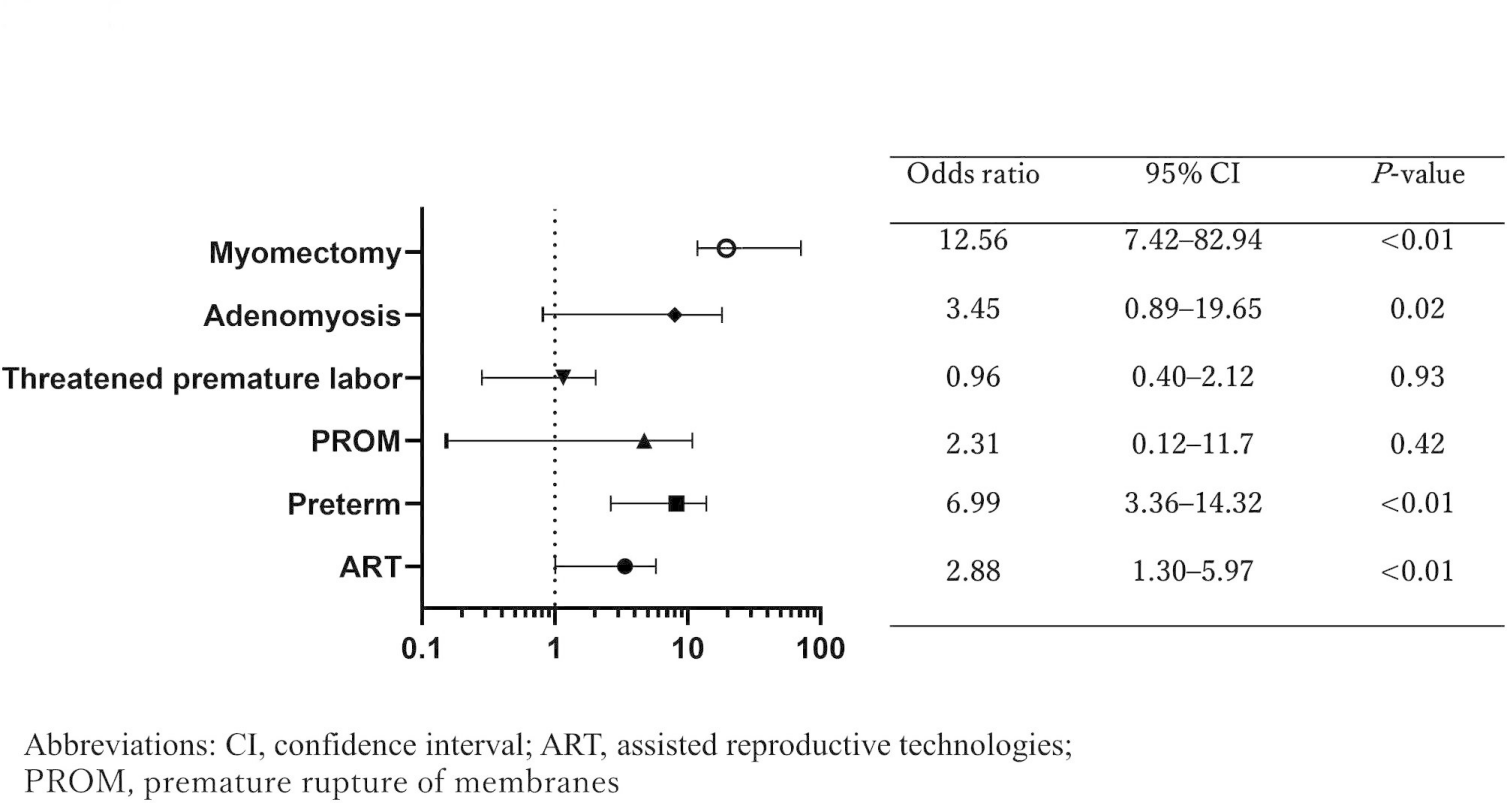
Risk factors for uterine rupture. Aadenomyosis (OR: 3.45; 95% CI: 0.89–19.65; P = 0.02) was identified as an independent risk factor, in addition to ART pregnancies (OR: 2.88; 95% CI: 1.30–5.97; P < 0.01), premature delivery (OR: 6.99; 95% CI: 3.36–14.32; P < 0.01), and uterine myomectomy (OR: 12.56; 95% CI: 7.42–82.94; P < 0.01)

**Fig. 3:**
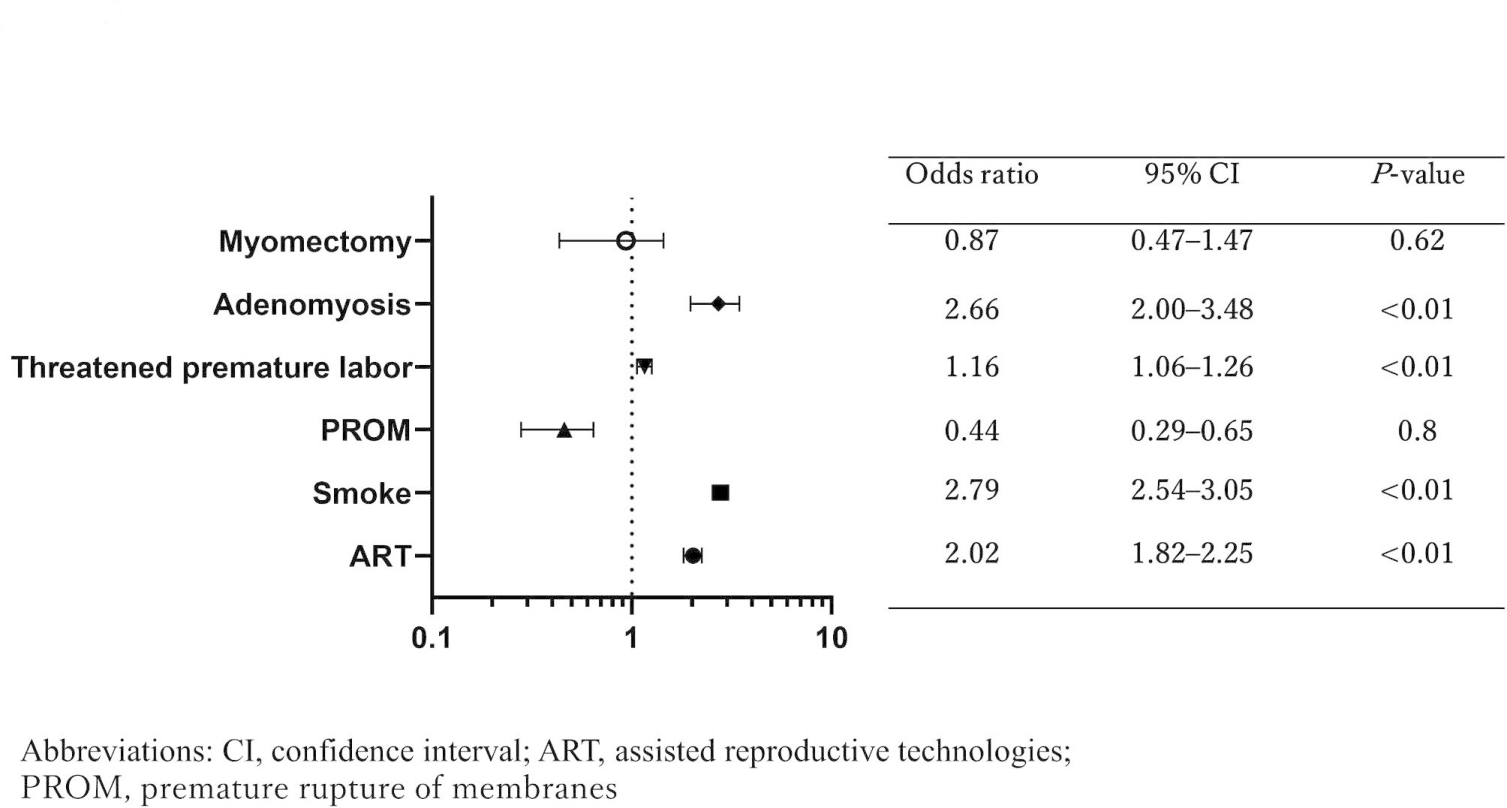
Risk factors for FGR. Adenomyosis was an independent risk factor for FGR (OR: 2.11; 95% CI: 1.27–3.31; P < 0.01)

**Fig. 4:**
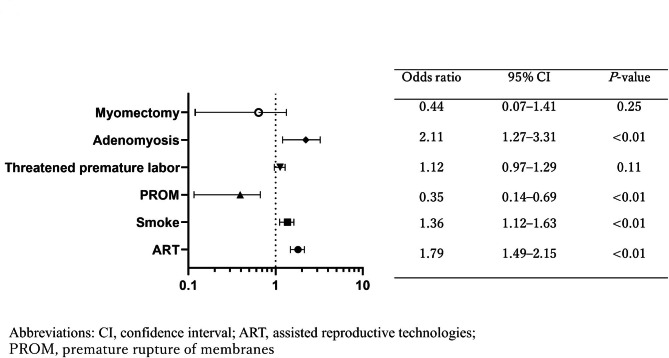
Risk factors for placental abruption. Adenomyosis was an independent risk factor for placental abruption (OR: 2.66; 95% CI: 2.00–3.48; P < 0.01)

A multivariate analysis identified ART pregnancies (odds ratio (OR): 7.20; 95% confidence interval (CI): 6.26–8.28; *P* < 0.001), complication of adenomyosis (OR: 2.26; 95% CI: 1.43–3.27; *P* = 0.001), and history of uterine myomectomy (OR: 2.62; 95% CI: 1.5–4.22; *P* < 0.01) as independent risk factors for placenta accreta (Fig. 1) [5]. In an examination of risk factors for uterine rupture, adenomyosis (OR: 3.45; 95% CI: 0.89–19.65; *P* = 0.02) was identified as an independent risk factor, in addition to ART pregnancies (OR: 2.88; 95% CI: 1.30–5.97; *P* < 0.01), premature delivery (OR: 6.99; 95% CI: 0.12–11.70; *P* = 0.42), and uterine myomectomy (OR: 7.41; 95% CI: 12.56–82.94; *P* < 0.01) (Fig. 2). Adenomyosis was an independent risk factor for placental abruption and FGR (OR: 2.11; 95% CI: 1.27–3.31; *P <* 0.01, and OR: 2.66; 95% CI: 2.00–3.48; *P <* 0.01, respectively) (Figs. 3 and 4).

## Discussion

### Principal findings

The present study investigated the impact of adenomyosis on perinatal outcomes using the JSOG Perinatal Registry Database and identified adenomyosis as a risk factor for placenta accreta as well as an independent factor for uterine rupture, placental abruption, and FGR. This indicates that perinatal management of pregnancies complicated by adenomyosis should be conducted considering these risks.

### Results in the context of what is known

Several studies have investigated the effect of adenomyosis on perinatal outcomes. Cozzolino et al. reported that pregnancies complicated by adenomyosis should be managed at a tertiary facility due to its adverse effect on perinatal outcomes, such as miscarriage, premature birth, and premature rupture of membranes [6]. In addition, adenomyosis requires due attention, as it may increase the risk of placental malposition and preeclampsia [7]. A previous Japanese study of 314 patients with adenomyosis reported an association of the condition with premature birth and low birth weight [8]. Similarly, Shin et al. reported that adenomyosis was a risk factor for premature birth and low birth weight, emphasizing the importance of ultrasound findings [9]. A previous study on a large cohort reported that the presence of endometriosis and adenomyosis significantly increased the prevalence of obstetrics complications, after adjusting for the influence of ART outcomes [4].

Few studies investigated the association between adenomyosis and placental position. A Japanese study reported an increased frequency of perinatal complications in cases where the placental formation was at the site of the adenomyosis lesion [10]. In particular, placenta previa was reported in 23.1% of endometriosis patients with severe adenomyosis [11]. In the present study, placenta previa was found in 7.8% (95/1204) of the cases, and the frequency may be higher in those with severe adenomyosis.

Several studies have examined the association between adenomyosis and uterine rupture. Vimercati et al. reported that adenomyosis diagnosed prior to pregnancy was associated with the risk of uterine rupture during delivery [12]. They stated that appropriate pre-pregnancy counseling is important and that patients with adenomyosis should be advised of the risk of uterine rupture. A nationwide survey in Japan on the incidence and prognosis of uterine rupture reported that uterine rupture during ongoing labor resulted in poor perinatal outcomes and particularly increased the frequency and risk of hysterectomy and cerebral palsy in newborns [13]. However, the frequency and risk of uterine rupture in these reports vary, as they may be affected by various environmental factors, such as the method of delivery management and mode of delivery at each facility, as well as timing of delivery.

Our present, large-scale study showed that adenomyosis increased the risk of uterine rupture as well as placenta accreta and that the incidence of uterine rupture was significantly higher in patients with adenomyosis than in those without adenomyosis. Furthermore, adenomyosis in pregnancies increases the frequency of placental abruption or FGR [4], and the present study, with far more than 300 cases, shows that it is an independent risk factor for both diseases.

On the other hand, although a previous Japanese study showed that adenomyomectomy was a risk factor for uterine rupture, the present study did not evaluate the presence or absence of adenomyomectomy. There have been few reports on the course of pregnancy after adenomyomectomy, and the management of adenomyosis is complex [14, 15]. Therefore, well-designed studies are needed in the future.

In recent years, there have been increasing number of reported adenomyomectomy cases. Zhou et al. reported that the use of the double-flap method for diffuse adenomyosis improved the prognosis of pregnancy [16]. Osada et al. and Nishida et al. have established their own surgical procedure for adenomyomectomy and reported a reduced risk of uterine rupture during pregnancy [17, 18]. A systematic review by Younes et al. stated that surgical treatment for refractory adenomyosis leads to improvement of symptoms and fertility. However, an optimal and definitive treatment has not been proposed, as there have been varying reports on pregnancy status after treatment [19]. The present study did not determine the effect of surgery since the presence or absence of adenomyomectomy could not be properly evaluated. However, surgery for adenomyosis likely increases the frequency and risk of perinatal complications, as is the case of myomectomy.

These findings suggest that adenomyosis increases the risk of premature birth, low birth weight, placental malposition, and uterine rupture. Therefore, pregnant patients with adenomyosis should be managed at an adequate perinatal facility.

### Clinical implications

It is important to understand that adenomyosis can be diagnosed only before conception or during early pregnancy, and the myometrium needs to be carefully examined by ultrasonography if adenomyosis is detected in early pregnancy. To our knowledge, there are no reports with data on > 1000 patients with adenomyosis. It is important to conduct clinical trials in which transvaginal ultrasonography and pelvic MRI are performed early in pregnancy, and tumor markers such as CA125 are measured, followed by evaluation of the pregnancy course.

### Research Implications

In future, we plan to conduct studies with higher accuracy, using multi-year databases, and to examine the perinatal outcomes of patients undergoing adenomyomectomy. With the social advancement of women and further popularization of infertility treatments, such as ART, pregnancies may face a variety of increased perinatal risks in the future, and procedures such as uterine surgery at a reproductive age should be performed with caution. In particular, further study will be conducted on the nature of infertility treatment, the number of ART procedures performed, history of miscarriage surgery and adenomyosis incidence, and obstetric complications.

### Strengths and Limitations

This study has some limitations. First, the location of lesions was unknown (anterior, posterior, or diffuse distribution), and details of severity were also unclear. As the effect of adenomyosis on delivery may be dependent on the location or extent of the lesion, further analyses are required. Second, the nature of relationship between placental malposition and location of adenomyosis lesion was unknown. If implantation occurs at an invasion site of the endometriosis lesion into the muscle layer, the risk of developing placenta accreta may be increased. To investigate this in detail, further studies of adenomyosis lesions and placental position are needed.

## Conclusions

The present study showed that pregnancies complicated by adenomyosis may be associated with an increased risk of developing placenta accreta, placental abruption, and FGR.

## Data Availability

All data generated or analysed during this study are included in this published article.

## Abbreviations

FGR: Fetus growth restriction
JSOG: Japan Society of Obstetrics and Gynecology
ART: Assisted reproductive technology
